# Towards a program of focused and applied curriculum research

**Published:** 2013-09-30

**Authors:** Marcel D’Eon

**Affiliations:** University of Saskatchewan, Saskatoon, Saskatchewan, Canada

## Abstract

Though hundreds of journal pages have been packed with studies describing, analyzing, and synthesizing the benefits of Problem-based Learning (PBL) over conventional curricula, we still don’t really know why. Currently it is impossible to say which of the various elements contributes to any incremental student learning. We need to apply the scientific method to studies of curriculum delivery. Accumulating evidence from strong studies in messy real-world situations will eventually yield important insights and instrumental truths for real medical schools that teachers and administrators can then implement. Examples of feasible experimental designs might include a factorial study. More effective curriculum development is possible only through a renewed applied research agenda that is both focused and grounded in the real world.

The last several decades have seen their fill of comparisons of conventional curricula with PBL.^1^–^8^ Hundreds of journal pages have been packed with studies and articles describing, analyzing, and synthesizing the purported, and sometimes marginal, benefits of PBL over conventional curricula. Even if we were confident that PBL produced better outcomes (and the jury still seems out on that question), we would not know why.^9^ Conventional curricula and PBL are in fact bundles of components with so many differences between them that it is impossible to say which of the various elements contributes to any incremental student learning, to what degree, or in what ways. For this reason Norman and Schmidt^10^ strongly discouraged real-world curriculum studies even calling them futile. They instead advocated for laboratory-style studies and structural equation modeling. I am obviously not the first to declare that we need to move beyond grand comparative studies but I do believe that well designed comparative curriculum studies in the messy real world will actually contribute to, and are necessary for, a productive program of inquiry into curriculum delivery.

The adoption and implementation of new approaches to curriculum delivery such as Team-based Learning (TBL),^11^ the flipped* classroom,^12^ and case-based instruction,^13^ together with PBL and of course the conventional predominantly lecture-based approach, will provide excellent opportunities for more and better comparative curriculum studies. It is not hard to imagine studies in a few years that compare PBL to TBL just as we have seen with PBL and conventional curricula. Given the close similarities between the two (small groups working cooperatively and independent preparation prior to working on cases and application exercises during class time), we will at least have isolated smaller bundles of components and be closer to identifying active ingredients in successful curriculum delivery approaches that eluded us when we had only PBL and conventional curricula.

Notwithstanding the improvement that these types of studies may bring, we will not have done enough. Why stop at comparing these approaches as indivisible units as we did for PBL and conventional curricula? We need to know more specifically what it is about PBL (or TBL or the flipped classroom) that works (or does not work), how much, why, and under which circumstances. We must isolate individual components of each method and test for a practically significant effect. This idea is not new but amplifies previous urgent appeals to apply the scientific method to studies of curriculum delivery.^9^,^10^ How else are we to answer the question “why” – to discover the causal components and the elusive active ingredients in various curriculum delivery approaches? However, unlike Norman and Schmidt,^10^ I think it is perfectly acceptable to conduct at least some of these in real medical schools. Granted, researchers may not be able to eliminate or control all the variables in real world environments but (chaos theory aside) we should be able to control the ones that generally matter, the ones that we know from theory and previous studies are likely to have a strong effect on important outcomes. Furthermore, medical schools must function in a real world and not in an education laboratory. Applied research studies with real curricula and real students in their actual environments are essential, like clinical trials of drugs developed in wet labs. Accumulating evidence from strong studies in messy real-world situations will eventually yield important insights and instrumental truths for real medical schools and real medical students that teachers and administrators can then implement.

Let me propose a few examples of possible studies. Based on what we know about self-directed learning,^14^ we can create and test a version of PBL wherein, keeping all other components intact as much as possible, first year students are *not* required to find their own materials to help them understand the case before them. We could isolate and test this self-directed learning component of PBL by providing students in our experimental group with relevant text and/or audio-visual resources that were carefully selected by the faculty, as is done in TBL or the flipped classroom, or by engaging them in an excellent focused lecture as they might get in case-based or conventional curricula. Or what might we find if, as a part of case-based learning, TBL, or the flipped classroom, based on what we know of instructional design,^15^ students in an experimental group engage with the case or problem as much as they are able before learning all the relevant material like they do for PBL?

Since many of the features of these approaches to curriculum delivery work in concert, researchers with access to sufficient numbers of participating medical students may want to consider more powerful factorial studies.^16^ Using the previous examples, a factorial design would have four cells, each one with a different combination of (1) student vs. teacher-directed knowledge acquisition as in TBL and (2) knowledge first then engagement with a problem vs. initial engagement prior to knowledge acquisition as in PBL. Table 1 contains a representation of this design. The analysis of various levels of outcome data would be able to tell us which, if any, of those two factors produced a main effect and which ones produced an interaction or moderator effect. We might find that initial engagement first followed by teacher-directed resources works best but we won’t know till we try. These examples are but a few of the hundreds of potential studies that, together, would form a focused, experimental, and applied research program in medical education curriculum delivery.

Grand curriculum studies that usually pitted PBL against conventional curricula, besides yielding few useful findings, have also forced curriculum planners into false dichotomies, a needlessly restricted either- or choice. The example studies I have suggested move us in the direction of more flexible and creative planning. From the flipped classroom and TBL I have suggested modifying classical PBL by introducing faculty resources for students to use when researching their learning issues. From PBL I have suggested modifying the flipped classroom and TBL by introducing an initial experience with the case or application exercise without complete prior preparation. Once we have the data from this new generation of studies we will be in a better position to be able to pragmatically re-bundle those elements and components of each approach that individually or in concert have the most impact on various outcomes into new more effective models instead of dogmatically advocating for one model of curriculum delivery or another (PBL, TBL, etc.). Such a creative and evidence-informed approach to curriculum development is possible through a renewed applied research agenda that is both focused on and grounded in the real messy world of medical schools.

## Figures and Tables

**Table 1:** Factorial study design of curriculum delivery approaches

Factors	Student directed knowledge acquisition as in PBL	Teacher-directed knowledge acquisition as in TBL or flipped classroom
**Knowledge first then problem as in case-based learning or TBL**	Students seek the knowledge first, then engage in the problem	Teacher provides materials then students engage in the problem
**Problem first followed by knowledge and then problem again as in PBL**	Students engage in the problem, seek knowledge, then tackle the problem again (classic PBL)	Students engage with the problem, teacher provides materials, then students re-engage with the problem
